# Severe skin toxicity during whole-brain radiotherapy, targeted therapy, and additional drug intake including St. John’s wort skin oil

**DOI:** 10.1007/s00066-020-01739-0

**Published:** 2021-01-24

**Authors:** Tanja Eichkorn, Fabian Schunn, Sebastian Regnery, Rami El Shafie, Juliane Hörner-Rieber, Sebastian Adeberg, Klaus Herfarth, Jürgen Debus, Laila König

**Affiliations:** 1 Department of Radiation Oncology, Heidelberg University Hospital, Im Neuenheimer Feld 400, 69120 Heidelberg, Germany; 2 National Center for Radiation Oncology (NCRO), Heidelberg Institute for Radiation Oncology (HIRO), Im Neuenheimer Feld 400, 69120 Heidelberg, Germany; 3 Clinical Cooperation Unit Radiation Oncology (E050), German Cancer Research Center (dkfz), Im Neuenheimer Feld 280, 69120 Heidelberg, Germany; 4 National Center for Tumor diseases (NCT), Heidelberg, Germany; 5 Deutsches Konsortium für Translationale Krebsforschung (DKTK), Partner Site Heidelberg, German Cancer Research Center (dkfz), Im Neuenheimer Feld 280, 69120 Heidelberg, Germany

**Keywords:** Radiosensitizer, Ramucirumab, Docetaxel, Folliculitis capitis, Radioimmunotherapy

## Abstract

**Background:**

Metastatic non-small cell lung cancer (NSCLC) often requires a multimodal treatment including chemotherapy, targeted therapy and radiotherapy. In addition to this, many patients take supportive drugs. Since only scarce data on possible interactions between radiotherapy and pharmaceutical or herbal drugs exist, description of clinical cases is of special interest.

**Case report:**

A patient with stage IV NSCLC was treated with docetaxel/ramucirumab followed by radiotherapy for brain and bone metastases while taking several other over-the-counter drugs (OTCs) including topical St. John’s wort skin oil.

**Results:**

A 63-year-old female patient with stage IV NSCLC presented with 11 asymptomatic brain metastases and a painful osteolytic bone metastasis in the 12th thoracic vertebral body (T12). Four weeks before the start of palliative whole-brain radiotherapy and bone irradiation of T12, she was administered a combination of docetaxel and ramucirumab. At an administered dose of 24 Gy, the patient presented with severe folliculitis capitis, while skin examination over the thoracolumbar spine was unremarkable although skin dose was similar. After thorough questioning, the patient reported using a herbal skin oil that contained St. John’s wort for scalp care only, but not for skin care of her back during radiotherapy. After stopping the topical application of the skin oil, folliculitis improved with a course of systemic and topical antibiotics within 10 days, though the healing process was prolonged and included desquamation and hyperpigmentation.

**Conclusion:**

St. John’s wort seems to be a significant radiosensitizer for photon radiotherapy and can cause severe skin toxicity even though the literature lacks data on this interaction. As an OTC, it is easily accessible and often used by oncological patients due to antidepressant and local antimicrobial and pain-relieving effects.

## Introduction

Metastatic non-small cell lung cancer (NSCLC) mostly requires a multimodal treatment. In recent decades, targeted therapies have become a well-established component of this multimodal treatment alongside the longer-established components chemotherapy and radiotherapy (RT). Several agents are taken into consideration, amongst them ramucirumab, an anti-angiogenetic human immunoglobulin G1 monoclonal antibody. Ramucirumab binds to the extracellular domain of the vascular endothelial growth factor (VEGF) receptor‑2 (VEGFR-2) with high specificity and affinity, thus blocking the interaction of VEGFR‑2 and VEGF ligands [1]. For patients with stage IV NSCLC, ramucirumab plus docetaxel was shown to improve survival as second-line treatment [2]. Due to the multimodality required for the treatment of metastatic NSCLC [3], simultaneous application of ramucirumab, docetaxel, and RT is common.

## Case report

The case of a female patient with stage IV NSCLC who received docetaxel/ramucirumab 1 month before the start of palliative whole-brain radiotherapy (WBRT) as well as bone irradiation for brain and bone metastases is reported here. Retrospective investigations revealed that the patient had also been taking several over-the-counter drugs (OTCs) during the course of RT.

## Results

A 63-year-old female patient was diagnosed with stage IV NSCLC in May 2019 and received a combination therapy of carboplatin, pemetrexed, and pembrolizumab. However, as the patient soon developed cerebral and osseous tumor progression, the treatment was changed to the combination of docetaxel and ramucirumab in April 2020. In May 2020, 12 months after the primary diagnosis, she presented to our outpatient clinic with 11 asymptomatic brain metastases and a painful osteolytic bone metastasis in the 12th thoracic vertebral body (T12) with infiltration of the spinal canal and consecutive reduced stability as well as local pain.

**Fig. 1:**
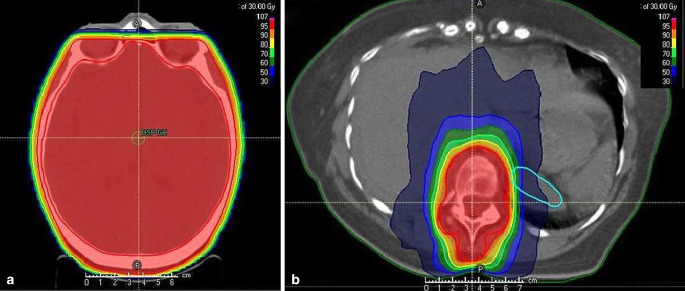
Representative transverse images from whole-brain radiotherapy (WBRT; **a**) and spinal irradiation (**b**) showing the treatment field and isodoses for both treatment plans

Therefore, palliative WBRT (single dose 3 Gy, total dose 30 Gy, 3D conformal RT) was started as well as bone irradiation of T12 (single dose 3 Gy, total dose 30 Gy, volumetric modulated arc therapy). Average skin dose was in both localizations approximately 70–80% of the planned 30 Gy. Representative images from the RT plans are depicted in Fig. 1a, b.

At the time, when cerebral and bone irradiation started, the patient had received one cycle of docetaxel and ramucirumab exactly 4 weeks earlier.

**Fig. 2:**
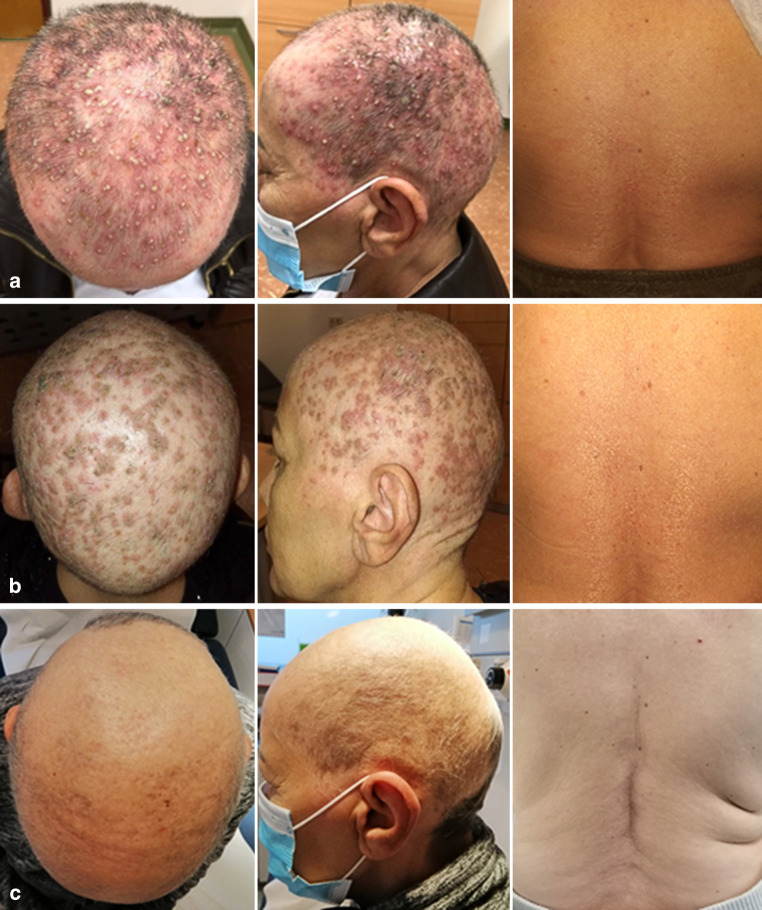
**a** Primary diagnosis of severe folliculitis capitis-like skin toxicity (CTCAE grade 3 dermatitis and folliculitis) during whole-brain radiotherapy (WBRT) without simultaneous skin toxicity during T12 irradiation (CTCAE grade 0). All photos were taken on the same day after the eighth fraction of both WBRT and T12 irradiation (cumulative dose 24 Gy for each WBRT and T12 irradiation). **b** 10-day follow-up of severe folliculitis capitis-like skin toxicity (CTCAE grade 2 dermatitis and hyperpigmentation) during WBRT without simultaneous skin toxicity during T12 irradiation (CTCAE grade 0), all photos were taken 10 days after finishing both WBRT and T12 irradiation. **c** 5-month follow-up of severe folliculitis capitis-like skin toxicity (CTCAE grade 1 hyperpigmentation) during WBRT without simultaneous skin toxicity during T12 irradiation (CTCAE grade 0), all photos were taken 5 months after finishing both WBRT and T12 irradiation

After the seventh fraction (21 Gy) of both brain and bone RT, the patient presented with mild erythema and scattered small spots on her scalp (skin toxicity CTCAE grade 1). Over the weekend and after the eighth fraction (24 Gy), the symptoms worsened considerably. The skin showed severe folliculitis capitis with generalized redness of the irradiated scalp and innumerable pustules filled with pus (skin toxicity CTCAE grade 3, Fig. 2a). She complained of severe pruritus and mild pain on her scalp. Dermatitis was strictly limited to the irradiated scalp, whereas the patient’s skin on her back (at level T12, bone irradiation) was without pathological findings (skin toxicity CTCAE grade 0, see Fig. 2a).

Detailed patient history revealed that she had been taking several OTCs without our knowledge and despite medical history assessment prior to start of RT. Due to invisible skin irritation including light pruritus and light local burning pain during chemotherapy, she was prescribed a herbal “analgesic skin oil” that was considered to be harmless by an oncologist. Therefore, the patient did not tell the radiation oncologist about that local treatment when RT was started. She applied it twice during radiotherapy: at the beginning and after worsening of the symptoms. This skin oil contained 98.6 vol% St. John’s wort, 0.009 vol% cajeput oil, 0.003 vol% lavender oil, and 0.003 vol% rosemary oil. Additionally, she was taking a combination of selenium, folic acid, vitamin D3, calcium, and magnesium.

Further assessment revealed a normal leucocyte count of 8.45/nl (reference value 4–10/nl) and a recently slightly elevated C‑reactive protein level of 10.8 mg/l (reference value <5 mg/l). Cotton swabs of pus samples for microbiological analysis to test for bacteria and fungi came back positive for *Staphylococcus epidermidis*. The skin oil topically used by the patient was wiped off as well and came back negative for bacteria and fungi.

Due to the excessive toxicity seen in Fig. 1, RT was paused immediately and the patient was treated orally with minocycline (50 mg, two times per day) and topically with nadifloxacin ointment (twice per day) as well as antibacterial undecylenamidopropyl betaine/polyhexanide (Prontosan®, B. Braun Melsungen AG, Melsungen, Germany) wound dressings. After 2 days, the general erythema and severe folliculitis-like skin toxicity improved markedly and we continued RT to ensure oncologic therapy. The folliculitis-like skin toxicity further improved within 12 days, although the healing process was prolonged and included desquamation and hyperpigmentation. At the follow-up visit after 5 months, the skin had healed well and presented with beginning hair growth although receiving chemotherapy at the time (for follow-up pictures after 10 days see Fig. 2b and after 5 months see Fig. 2c). Oncologically, the patient is in partial remission under continuing chemotherapy.

## Discussion

A patient who had been previously treated with chemoimmunotherapy followed by RT was simultaneously irradiated at two different locations with the same technique and target volume dose, and a similar skin dose. After 24 Gy, she presented with severe skin toxicity at one location but without any skin toxicity at the other location. Discrepancy in skin reactions at the two simultaneous irradiation targets provided important hints for pathogenetic differentials.

Due to the patient’s history of recent docetaxel and ramucirumab administration, interaction between radiotherapy and combined targeted therapy and chemotherapy can be suspected as a possible cause for the present disproportionately severe skin toxicity [4–6]. For bevacizumab, another VEGF inhibitor, several clinical trials describe the following toxicities during simultaneous radiotherapy:

The AvaGlio trial, which applied bevacizumab simultaneous to a 60 Gy (2 Gy single dose) RT in glioblastoma patients, describes the most commonly observed adverse events as mucocutaneous bleeding, thromboembolic events, hypertension, proteinuria, and thrombocytopenia [7]. According to a randomized phase II trial comparing bevacizumab plus carboplatin and paclitaxel with carboplatin and paclitaxel alone in advanced NSCLC, bleeding was the most prominent adverse event, with mainly minor mucocutaneous hemorrhage and major hemoptysis [8]. Nevertheless, there are some case reports of simultaneous bevacizumab and stereotactic body radiotherapy (SBRT) with 45 Gy in three fractions where life-threatening bleeding occurred and an association of increased toxicity when combining both modalities cannot be ruled out [9]. A meta-analysis on bevacizumab toxicity mainly found, among others, hematological disorders, wound-healing disorders, and, rarely, gastric ulcers [10]. Overall, bevacizumab toxicity seems to be more frequent when combined with high single doses in hypofractionated radiotherapy/SBRT. Skin toxicity, however, seems to be very uncommon for VEGF inhibitors.

Nevertheless, the therapy with docetaxel/ramucirumab was started just 4 weeks before RT and various targeted therapies are well-known to cause (e.g., acne-like) skin toxicity [11, 12]. For ramucirumab, skin toxicity has been described, including especially palmar-plantar erythrodysesthesia syndrome but without reports of increased rates of folliculitis [13]. Furthermore, several targeted therapies show prolonged biological half-life, so that possible interaction with RT may also occur weeks after immunotherapy. For ramucirumab, the mean population estimate for half-life is 13.4 days [14], so our patient still had about a quarter of the initial dose of ramucirumab in her body when radiotherapy was started.

Nevertheless, there are counterarguments for that theory. The severe folliculitis capitis-like skin toxicity was strictly limited to the WBRT region, whereas the second RT localization at T12 demonstrated no skin toxicity at all, which is highly uncommon in immunotherapy-related skin toxicity during RT. Skin dose was similar in both localizations (Fig. 1).

Due to the discrepancy in skin reaction between the two RT localizations as described, a genetic radiosensitivity, which can be found in 1–5% of the population [15], seems to be unlikely as well.

For these reasons, other possible causes of irradiation-induced skin toxicity should be taken into consideration and discussed. The simultaneous use of OTCs needs to be considered as well. Especially the skin oil that was applied locally in the RT field can be suspected to have phototoxic properties because the toxicity was very limited to skin areas that were exposed to both skin oil and irradiation treatment, even if the patient applied the oil only twice during RT. St. John’s wort (with the pharmaceutical ingredient hypericin) is well known to cause phototoxic side effects if ingested orally. There are even trials published that investigate whether hypericum-assisted photodynamic therapy can be used for anaplastic thyroid cancer [16]. With topical application of St. John’s wort, a significant increase in the development of erythema can be observed after sun exposure [17]. Available data reported erythematous skin reactions but not folliculitis-like skin toxicity after St. John’s wort topical application and sun exposure. A case report published in 2001 indicated that oral application of St. John’s wort caused severe erythema and large blisters after laser therapy for solar keratosis [18]. Nevertheless, both sun exposure and laser therapy cannot be directly compared to photon irradiation, during which severe folliculitis capitis-like skin toxicity occurred in our patient. The literature reports one case describing radiation recall dermatitis that started 11 months after finishing radiotherapy as a result of taking hypericin [19]. But there are no data available that investigate acute skin reactions due to St. John’s wort during or shortly after radiotherapy. Paradoxically, despite the well-known phototoxicity with sunlight of St. John’s wort, a study exists that suggests that the use of skin oil containing St. John’s wort helped to resolve irradiation-induced skin toxicity during radiotherapy of head and neck cancers. This study was a single-arm observational trial without a comparison group including 28 patients, and therefore the suitability of the study design for assessing drug effectivity can be questioned [20]. Our patient used a skin oil that additionally contained cajeput oil, lavender oil, and rosemary oil, which have antimicrobial properties but may also be sensitizing [21]. In addition, the application of lipids may have occluding effects on hair follicles and therefore may favor the development of folliculitis. Hair follicles on the scalp skin differ from hair follicles on back skin, which might make the scalp skin more vulnerable to folliculitis capitis. Due to this, the literature recommends moisturizing rather than greasing topicals for skin care during radiotherapy, even if no gold standard for skin care during radiotherapy exists [22].

In summary, the cause of the severe skin toxic reaction on the patient’s scalp was most probably multifactorial, but also emphasizes the influence of locally used St. John’s wort skin oil.

## Conclusion

As an OTC, St. John’s wort is easily accessible even without physicians’ knowledge and is often used by oncologic patients due to antidepressant effects upon systemic application and antimicrobial as well as anti-inflammatory effects upon topical application.

Additional medications, often applied without knowledge of the treating physicians, may cause severe side effects when combined with radiotherapy. This case demonstrates the importance of close clinical observation of patients receiving additional chemotherapy and targeted therapy simultaneously to RT, as well as the necessity of obtaining information of additional medications when combined with RT. Especially when prescribed as “rather harmless” herbal medications, radiation oncologists should stay alert and obtain information of possible radiosensitizing ingredients. St. John’s wort oil seems to be a significant radiosensitizer for photon radiotherapy and can cause severe skin toxicity, especially when combined with other sensitizing and skin-irritating substances in a multifactorial setting, even though the literature is lacking in data on this interaction.
